# Benchmarking nanopore-based strategies for antimicrobial resistance prediction

**DOI:** 10.1099/mgen.0.001885

**Published:** 2026-11-03

**Authors:** Natalie Ring, Alison S. Low, Rhodri Evans, Marianne Keith, Gavin K. Paterson, David Gally, Tim Nuttall, Dylan Neil Clements, J. Ross Fitzgerald

**Affiliations:** 1 The Roslin Institute, University of Edinburgh, Edinburgh, UK; 2 The Royal (Dick) School of Veterinary Studies and the Roslin Institute, University of Edinburgh, Edinburgh, UK

**Keywords:** AMR, AST, *in silico* prediction, metagenomics, nanopore sequencing

## Abstract

Antimicrobial resistance (AMR) presents a pressing need to ensure that the right antimicrobials are used to target the right microbes at the right time. Ideally, the appropriate antimicrobial is selected after patient samples have been cultured and assessed with antimicrobial sensitivity testing (AST). However, the time needed for culture-based diagnosis leads to immediate empirical treatment, often with broad-spectrum and/or high-tier antimicrobials. Direct nanopore metagenomic whole-genome sequencing to identify pathogens and predict their AMR is a rapid and patient-side alternative. A limitation of this approach is potential inconsistencies in i predicted AMR phenotypes. Here, we benchmarked the current performance of *in silico* AMR prediction strategies for nanopore-generated long-read data. Using real-world nanopore data paired with AST phenotyping for 201 samples representing 27 bacterial species, we assessed the impact of basecalling mode, data volume and assembly strategy and compared the performance of eight *in silico* AMR prediction tools with seven AMR databases. We found that basecalling accuracy mode does not significantly affect the overall accuracy of *in silico* AMR predictions but assembly strategy and data volume both do. Prediction tools using the ResFinder database scored best for balanced accuracy (0.80±0.02 for both ResFinder and ABRicate), whilst DeepARG scored best for sensitivity (0.65±0.03); predictions were more accurate for some antibiotic classes and genera than others. However, even the best-performing *in silico* AMR prediction strategy missed some resistance identified by lab-based AST. We conclude, therefore, that currently *in silico* AMR prediction can supplement lab-based AST but cannot yet replace it.

**Impact Statement**

Antimicrobial resistance (AMR) is threatening modern standards of human and veterinary healthcare. Rapid and patient-side diagnostic tests are needed to diagnose bacterial infections and allow clinicians to select effective antibiotics. Current tests based on bacterial cultures take several days, which may delay diagnosis and treatment or lead to inappropriate ‘just in case’ treatment while waiting for the results. In contrast, nanopore metagenomic whole-genome sequencing can identify bacterial infections and predict which antibiotics will be effective in minutes to hours. However, the accuracy of these tests is uncertain. We, therefore, compared the performance of eight AMR prediction tools and seven databases of AMR determinants, using 201 bacterial samples with known antibiotic susceptibility and/or resistance. We found that the sensitivity (i.e. true positive rate), specificity (i.e. true negative rate) and overall accuracy of the tools and databases varied. In particular, even the best-performing AMR prediction methods missed some AMR. Therefore, while these tools are useful for rapid and patient-side diagnosis and treatment decisions, they still have limitations and should be used alongside bacterial cultures and antibiotic sensitivity testing.

## Data ummary

Sequencing data for the samples sequenced for this study are available at the National Center for Biotechnology Information under BioProject ID PRJNA1292816 (sequence read archive (SRA) accessions for all datasets used here are available in Table S1, available in the online Supplementary Material).

Commands and code used can be found at: https://github.com/nataliering/nanopore_AMR_tools/

The authors confirm all supporting data, code and protocols have been provided within the article or through supplementary data files.

## Introduction

Antimicrobial resistance (AMR) is an immediate and ongoing global threat that will impact health, food security and economic development [1, 2]. Over eight million annual AMR-associated deaths are forecast by the year 2050 [3]. Inappropriate and indiscriminate antimicrobial use exerts a selection pressure favouring the development and dissemination of bacterial AMR [4]. To avoid this, we need to use the right antimicrobial for the right infection at the right time. This is a one health challenge that encompasses human, production animal and companion animal healthcare.

Faster diagnosis to confirm a bacterial infection and determine its antimicrobial susceptibility will improve antimicrobial stewardship. The current gold-standard methods involve culturing samples to identify the bacteria, followed by antibiotic sensitivity tests (ASTs). Commonly used ASTs include disc diffusion, minimum inhibitory concentration (MIC) test strips and broth microdilution assays [5, 6]. These tests require a minimum of 16 h, but it often takes significantly longer to obtain the results. Immediate empirical antimicrobial treatment may be unnecessary (where there is no bacterial infection) and/or may employ unnecessarily broad-spectrum or high-tier antimicrobials through fear of compromising patient outcomes. Excessive and inappropriate antimicrobial use is a major driver of AMR [4]. In addition, culture-based ASTs are effectively ‘a sample of a sample’ (i.e. patient sample – culture – culture sample – AST) and favour less fastidious bacteria. AST results may not, therefore, reflect the whole bacterial population and antimicrobial susceptibility patterns in the patient’s infection.

Metagenomic whole-genome sequencing (mWGS) using Oxford Nanopore Technologies’ (ONT) long-read sequencers is a rapid culture-free technique that involves the extraction and sequencing of all genomic DNA (gDNA) in a sample [7]. This contrasts with targeted approaches, such as 16S rRNA gene amplicon sequencing, which sequence only small portions of the genome [8]. The resulting DNA data from mWGS can be used to identify pathogens and to detect genes and mutations associated with AMR in as little as 10 min [9, 10]. Progress on developing mWGS-based rapid diagnostics pipelines has accelerated in recent years, and ONT’s platforms have been used to pilot rapid diagnostic methods for mWGS from clinical samples, such as sputum, respiratory fluid, blood, urine and skin swabs [11–26].

Robust, reliable and reproducible bioinformatic tools and pipelines will be vital to implement rapid mWGS-based diagnostics [27]. Accurate genomic detection of AMR determinants is also important beyond direct mWGS diagnostics, including isolate-based clinical and public health surveillance, outbreak investigation and environmental monitoring.

In 2020, a multi-laboratory study found wide discordance between the AMR phenotypes predicted by different laboratories using the same genomic datasets [28]. This discordance occurred as a direct result of differences in how each laboratory chose to process the data and the bioinformatic tools and databases used for the AMR predictions. Similarly, a comparison of tools to identify AMR from Illumina short-read datasets showed wide variability in their sensitivity, specificity and balanced accuracy [29]. No single tool performed equally well across every antibiotic class; some performed very well in terms of sensitivity but relatively poorly in terms of specificity.

Here, we analysed the performance of eight different AMR detection tools (ABRicate [30], StarAMR [31], ResFinder [32], AMRFinderPlus [33], c-SSTAR [34], resistance gene identifier (RGI) [35], DeepARG [36] and AMR++ [37]) on datasets produced using ONT sequencers. We included tools which require the assembly and annotation of sequencing reads into consensus contigs, as well as read-based tools. The tools use different methods to detect AMR determinants, including *k*-mer-based alignment, Hidden Markov Models and deep machine learning. Some tools offer a choice of AMR databases (e.g. ABRicate) whilst others are based around their own database (e.g. ResFinder, DeepARG).

To test the tools, we sourced a collection of 201 ONT datasets with matched lab-based AST phenotypic AMR profiles, representing 27 different bacterial species. Rather than restricting the benchmark to data generated using a single sequencing workflow, we made use of appropriate nanopore datasets identified through a literature search, which spanned different sequencing chemistries, basecalling approaches and source studies. This reflects the rapid technological development of ONT sequencing chemistry and the corresponding heterogeneity of the substantial body of existing nanopore data that remains available in online repositories for potential reuse. Using this dataset, we evaluated the effects of methodological variables, including sequencing chemistry, basecalling mode, data volume, assembly strategy, read- versus assembly-based prediction, tool/database choice and species-specific prediction. This design, therefore, allowed us to assess the robustness of AMR prediction patterns across the heterogeneous nanopore data landscape.

The primary benchmark comprised 151 monocultured isolate datasets. Accordingly, the principal tool performance comparisons reported here derive from the isolate sequencing data. A further 50 mixed or host-contaminated datasets were analysed separately as a secondary analysis, rather than pooled with the isolates, to assess whether broad performance patterns were also observed in more complex sequencing datasets representative of those likely to be encountered in a diagnostic context. We also compared the performance of tools at the level of antibiotic class and individual antibiotics.

## Methods

### Data collection and curation

A National Center for Biotechnology Information (NCBI) PubMed database search was conducted in May 2025, using various combinations of the terms ‘nanopore’, ‘AMR’, ‘MinION’ and ‘AST’. The resulting literature hits were manually reviewed to determine if (1) their raw sequencing data were publicly available and (2) their datasets included *in vitro* antimicrobial sensitivity testing (AST) results. If a paper fulfilled both of the above criteria, the relevant datasets were downloaded, and the following metadata was recorded for each sample (full data in Table S1): sample accession, sample type, MinION flow cell version, library preparation kit, basecaller used (and, where available, version and accuracy mode), species identified in original publication, AST results and original publication reference.

### Comparing monocultured isolates with metagenomic samples

For clarity, we use the term isolate whole-genome sequencing (WGS) to refer to sequencing of monocultured bacterial isolates and mWGS to refer to untargeted sequencing of gDNA from samples containing multiple organisms and/or host DNA. The datasets gathered here were divided into two groups: 151 monocultured isolates, which formed the primary benchmark, and 50 mixed or host-contaminated datasets, which were analysed separately as a secondary sample set. The monoculture group (151 samples) was used for the majority of the analysis described below, including comparison of basecalling and assembly strategies, investigation into data volume, comparison of tool and database performance, comparison of prediction accuracy across different antibiotic classes and comparison of prediction accuracy across different genera. The metagenomic group (50 samples) was used to assess whether the tool performance patterns identified by the monoculture analysis were also observed for the more ‘difficult’ sample types. Phenotypic AST results for all datasets, including mixed or host-contaminated datasets, were obtained from their corresponding source studies. In general, phenotypic AST results for metagenomic samples are generated by performing AST on isolates recovered by culture, while sequencing is performed on the original metagenomic sample (e.g. 11, 14).

### Read preparation

Data were downloaded in FASTQ format from the NCBI Sequence Read Archive using fastq-dump v2.11.3 [38]. NanoStat v1.6.0 [39] was used to determine mean read length, number of reads and overall number of bases. Datasets were excluded if their mean read length was less than the standard nanopore quality cut-off of 1,000 bp [40–43] or their overall number of bases was less than 25 Mb (around 5**×** coverage of the ‘average’ monocultured bacterial genome [44–47]). A set of 64 additional isolates (Table S2) were sequenced specifically for this study, representing species not otherwise found in the above literature search. See Supplementary Materials SM1 for full DNA extraction, library preparation and sequencing methods and Tables S1 and S2 for sample accessions and basecaller details.

Porechop v0.2.4 [48] was used to trim adapters from the FASTQ read sets which passed quality control (QC). For all datasets, including the mixed/metagenomic datasets, Filtlong v0.2.0 [40] was used to filter longer read sets (>0.5 Gb) down to 0.5 Gb, retaining the longest and highest quality FASTQ reads. Read sets already below 0.5 Gb were not filtered with Filtlong. Where an AMR tool based its predictions on raw FASTQ reads alone, the output Filtlong reads were used. Table S1 shows full sample metadata, including original data volume and whether each sample was filtered.

### Genome assembly and draft annotation

The trimmed reads were assembled with two different long-read assembly tools, Minimap2 v2.24-r1122/Miniasm v0.3-r179 [49, 50] and Flye v2.9 [51]. The Flye assembly was polished using Medaka v2.1.0 [52]. As full basecalling model metadata was not reported in the original publications for all samples in the dataset, the medaka_consensus command was run with automatic model selection enabled by omitting the -m parameter. Where the basecalling model could not be identified from the input data, Medaka would have used its default consensus model. The optional bacterial methylation polishing mode (--bacteria) was not enabled.

The Flye, Miniasm and Medaka assemblies were then all annotated with Prokka v1.14.6 [53]. All three assemblies (Miniasm, Flye and Medaka), along with the raw reads and annotations where applicable, were used to assess the performance of the tested AMR tools to determine the optimal assembly strategy. More details about each assembly tool can be found in the **Supplementary Materials** (**SM2**).

### AMR tools and databases tested

We tested a variety of assembly- and read-based tools, with every relevant database available to each tool. The tools and databases are summarized in Table 1 and more details are in the Supplementary Materials (SM3). Commands used for each tool are available from: https://github.com/nataliering/nanopore_AMR_tools/.

**Table 1.** AMR detection tools and databases tested here

Tool	Version	Resistance database(s)	Resistance database version	Input file(s)
ABRicate [30]	1.0.1	NCBI [33], CARD [35], ResFinder [32], ARG-ANNOT [121], MEGARes 2.0 [122]	14 Jan 2025	Assembly
AMRFinderPlus [33]	4.0.23	NCBI	2025-03-25.1	Assembly and annotations
ResFinder [32]	4.7.2	ResFinder and PointFinder	2.6.0 and 4.1.1	Reads or assembly
c-SSTAR [34]	2.1.0	ResGANNOT	na	Assembly
RGI [35]	6.0.4	CARD	4.0.1	Assembly, or assembly and annotations
StarAMR [31]	0.11.0	ResFinder	2024-08-06	Assembly
DeepARG [36]	1.0.2	DeepARG DB	na	Reads, or assembly and annotations
AMR++ [37]	3.0.6	MEGARes	na	Reads

AMR, antimicrobial resistance; ARG-ANNOT, Antibiotic Resistance Gene-ANNOTation; CARD, Comprehensive Antibiotic Resistance Database; DB, database; NA, not available; NCBI, National Center for Biotechnology Information; RGI, Resistance Gene Identifier.

For tools which used assembled contigs for AMR prediction, the Miniasm, Flye and Medaka assemblies were all tested with each tool/database combination. See Tables S3a and S3b for a full list of the 42 tool/database combinations, as well as the parameters applied by these tools.

Some of the tools tested here, such as ResFinder, offer species-specific AMR prediction for a limited number of species, facilitating the detection of AMR caused by species-specific point mutations. Where a sample was known to contain one of these species, all possible combinations were also trialled with this mode. Table S4 shows which tools were used with which species. Metagenomic samples containing multiple species were not included in the species-specific tests.

HAMRonization v1.1.9 [54] was used to convert the results of all tools into a consistent tabular form. See Supplementary Methods SM4 for details of the additional steps which were taken to ensure standardization of the way results were reported across different tools.

### Calculating performance metrics

For each sample, the original AST results were collated to create a per-antibiotic ‘phenotypic AMR’ table (Table S5). In addition, the Comprehensive Antibiotic Resistance Database (CARD) ontology [35] was used to assign antibiotics in our phenotypic AMR table to their drug class: aminoglycosides, beta-lactams, fluoroquinolones, glycopeptides, macrolides–lincosamides–streptogramins (MLS), phenicols, sulfonamides, tetracyclines, trimethoprim (diaminopyrimidines) and polypeptides. Rarer antibiotic classes were excluded from analysis due to insufficient numbers of resistant samples in our dataset. Table S6 shows which antibiotics were included in each class and which classes/antibiotics were excluded. A second table was then produced, which listed each sample’s phenotypic AST results in terms of drug class (Table S7). For each sample in our phenotypic AMR tables, each antibiotic or class was assigned ‘R’ (resistant), ‘S’ (susceptible) or ‘NA’ (not tested) based on the AST results from the original publications. Where a result in the phenotypic AMR table was ‘I’ (Intermediate), it was considered to be resistant.

Each set of results was then analysed at the level of specific antibiotic and antibiotic class. The tool results were compared with our phenotypic AMR table, and each antibiotic/class was scored as true positive (TP), false positive (FP), true negative (TN) or false negative (FN) for each sample. A TP occurred when both the phenotypic AMR result and the *in silico* tool prediction were ‘R’ for an antibiotic/class for that sample. A FP occurred when the phenotypic result was ‘S’ but the tool predicted ‘R’. A TN occurred when both the ground truth result and the tool prediction were ‘S’. A FN occurred when the phenotypic result was ‘R’ but the tool predicted ‘S’. Where an antibiotic or class was not AST tested for a sample (‘NA’), that antibiotic or class was excluded from the results comparison.

Performance metrics were then calculated for each tool according to the equations shown in Table 2.

**Table 2.** Equations used to calculate our performance metrics

Metric	Equation
**Sensitivity**	$\frac{TP}{TP+FN}$
**Specificity**	$\frac{TN}{TN+FP}$
**Precision**	$\frac{TP}{TP+FP}$
**Balanced accuracy**	$\frac{sensitivity+specificity}{2}$

Precision was considered undefined whereTP+FP=0.

FN, false negative; FP, false positive; TN, true negative; TP, true positive.

### Statistical analysis and data visualization

All statistical analyses were performed in Python v3.10.14 using pandas v2.3.0 [55, 56], SciPy v1.15.3 [57] and scikit-posthocs v0.11.4 [58]. Performance metrics were calculated for each comparison group as described above. Normality was assessed using the Shapiro–Wilk test [59] implemented with scipy.stats.shapiro, and homogeneity of variance was assessed using Levene’s test [60] implemented with scipy.stats.levene. Because the performance metrics were bounded between 0 and 1 and did not consistently meet parametric assumptions, non-parametric tests were used for group comparisons. Overall differences among groups were assessed using Kruskal–Wallis tests [61] implemented with scipy.stats.kruskal. Where Kruskal–Wallis tests indicated significant differences, pairwise post-hoc comparisons were performed using Dunn’s test [62] implemented with scikit_posthocs.posthoc_dunn, applying Bonferroni correction to account for multiple pairwise comparisons. Statistical significance was defined as *P*<0.05.

Approximate 95% confidence intervals (CIs) for mean sensitivity, specificity and balanced accuracy were calculated using the normal approximation (mean±1.96×standard error (SE)), with interval limits constrained to the range 0–1.

Figures were generated in R v4.1.2 using ggplot2 v3.3.6 [63], readxl v1.4.0 [64], dplyr v1.0.9 [65], tidyr v1.2.0 [66], stringr v1.4.0 [67], forcats v1.0.0 [68] and ggpubr v0.4.0 [69]. Summary tables were imported using readxl::read_excel and plotted using ggplot2. Bar plots were produced with geom_bar, error bars with geom_errorbar and heatmaps with geom_tile. Multi-panel figures and shared legends were arranged with ggpubr::ggarrange, and Dunn’s post-hoc significance annotations were added using ggpubr::stat_pvalue_manual. Final figures were exported using ggplot2::ggsave.

### Comparing super accuracy, high accuracy and fast basecalling mode

A subset of 64 monoculture samples (all those for which raw data were available, Table S2) were used to conduct a comparison of the accuracy of AMR prediction when using different basecalling accuracy modes. Where necessary, the unbasecalled FAST5 files were converted into pod5 format using pod5 v0.2.15 [70]. Dorado v0.9.6 was used for the R9.4.1/SQK-RBK004 datasets, with fast, high accuracy (HAC) and super accuracy (SUP) models v3.4, v3.3 and v3.6, respectively. Dorado v1.0.2 was used for the R10.4.1/SQK-RBK114.24 datasets, with model v5.2.0 for all three accuracy modes [71]. Full basecaller models are listed in Table S2 for each accuracy mode. Basecalling was performed using an NVIDIA A100 80 GB PCIe GPU. This created three basecalled read sets per sample (one each for fast, HAC and SUP). Each read set was processed through the data preparation, AMR prediction and analysis pipeline as described above and performance metrics were stratified by basecalling accuracy mode.

### Assessing the effect of data volume on AMR prediction accuracy

A subset of 61 samples (all those with at least 500 Mb of starting data, Table S8) were used to assess the effect of initial data volume on the eventual accuracy of AMR prediction. Each read set was processed through the data preparation, AMR prediction and statistical analysis pipeline as above, except that instead of filtering to 500 Mb with Filtlong, the read sets were filtered to 250, 100, 50 and 25 Mb. Including the read set originally filtered to 500 Mb, this produced five different sets of AMR predictions, representing 100**×**, 50**×**, 20**×**, 10**×** and 5**×** coverage of the ‘average’ length bacterial genome [44]. The performance metrics for each level of filtering were compared in the same way as the basecalling modes, as described above.

### Comparing older and newer sequencing data

The performance metrics for a subset of 28 samples (Table S9) were used to compare the accuracy of AMR predictions made from older and newer sequencing data. Fourteen samples sequenced with R10.4.1 chemistry and basecalled with Dorado were compared with 14 samples sequenced with R9.4/R9.4.1 chemistry and basecalled with Albacore or Guppy. The samples were species-matched between R10 and R9 as far as possible, according to the samples available in our dataset: five *Enterococcus*, three *Pseudomonas* and six *Staphylococcus* samples for each chemistry.

## Results

### A curated dataset of 201 samples to evaluate AMR prediction tools

A total of 201 samples met our selection criteria of having both ONT reads and AST phenotypes available, as well as passing our QC process [14, 72–84]. Table S1 gives the full metadata of all analysed samples and Table S10 gives the references of the original papers. The samples were sequenced on a variety of different flow cell types (R9.4.1, R9.5, R10.4.1), sequencing libraries were prepared using a variety of kits (for example, SQK-RPB004), and basecalling was performed with a variety of different basecallers (Albacore, Guppy, Dorado) and basecaller software versions. Starting volumes of data ranged from 28 to 16.5 Gb, with a mean of 662.51 Mb, median of 175.90 Mb and standard deviation (SD) of 2031.42 Mb. Mean read length per dataset ranged from 1.01 to 11.12 kbp, with a mean of 4.43 kbp, median of 4.00 kbp and standard deviation of 1.87 kbp. 151 samples were monocultured isolates, while 50 contained multiple species and/or potential host contamination. The monocultured isolates were used for the majority of the tests here; the remaining 50 were used to test how the tools performed with more ‘difficult’ samples.

Twenty-seven bacterial species were included in the monoculture or metagenomic datasets, with *Escherichia coli* the most common species (40 samples) and *Staphylococcus* the most widely represented genus (40 samples across 7 species). On average, each species was represented by 6.3 samples (range=1–40 samples). For the purposes of statistical power, only antibiotic classes recorded as resistant in ASTs from at least 5 (~2%) of our samples were considered (Table S5). Of these, beta-lactam resistance was most common (52.24% of samples). The mean number of samples in our dataset resistant to each antibiotic class was 35.7 (sd=30.11). See Table S4 for full species and AST details.

We also assessed the distribution of positive and negative AST calls. Overall, at the level of individual antibiotic there were 1,427 AST calls, 38% positive (resistant or intermediate) and 62% negative (susceptible), whilst at the level of antibiotic class there were 760 AST calls, 46% positive and 54% negative, suggesting acceptable overall balance. Tables S11 and S12 show the distributions across each antibiotic and class, with some antibiotics or classes more evenly distributed than others. Balanced accuracy was used instead of accuracy in subsequent analyses here, because it averages sensitivity and specificity and, therefore, reduces the influence of imbalance.

### Neither basecalling accuracy mode nor sequencing chemistry significantly affects overall AMR prediction accuracy

A subset of 64 monocultured samples were basecalled with Dorado in three different accuracy modes: fast, HAC and SUP. Each set of reads was then processed through the rest of the assembly, analysis and statistics pipeline and the results were stratified by basecalling accuracy.

Fig. 1 shows that no significant differences were found between the accuracy modes across any of the performance metrics, although there is a slight trend towards increased sensitivity from fast to HAC to SUP. All samples in our dataset were, therefore, carried forward for further analysis, regardless of original basecalling accuracy.

**Fig. 1.**
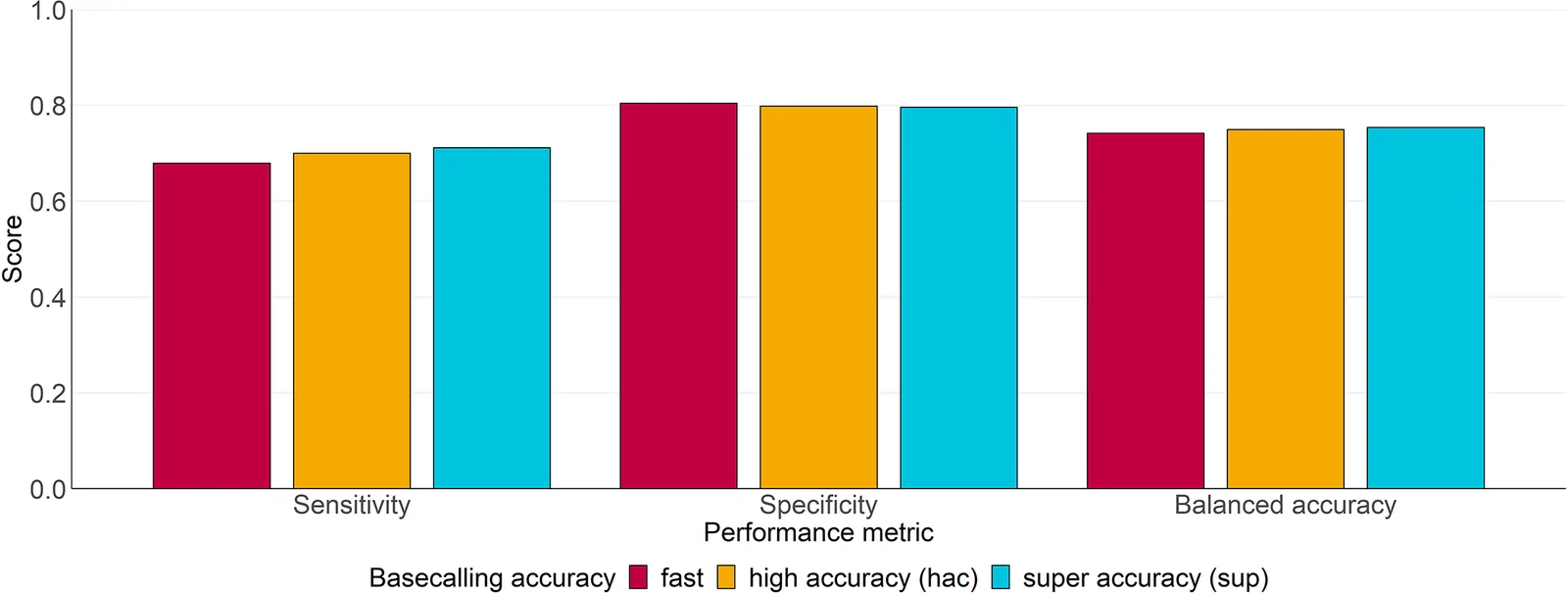
Performance metrics of our 64-sample subset which were basecalled with three different basecalling accuracy modes in Dorado. Metrics were calculated across all tools and all samples, then stratified by basecalling accuracy; fast, HAC and SUP. No significant differences were observed, according to the Kruskal–Wallis test.

The results for a small subset of species-matched R10.4.1 and R9.4/R9.4.1 were compared to reveal any differences due to the use of older or newer sequencing chemistry or basecaller. No significant differences were seen in any performance metric between R10 and R9.

### Flye+Medaka produces the best balanced accuracy, whilst reads-only produce the best sensitivity across all tools

Performance metrics were calculated for all 151 monocultured samples across all tool/database combinations, then stratified by assembly strategy (‘flye’, ‘medaka’, ‘miniasm’ and ‘reads’). As shown in Fig. 2, although no tool performed significantly better across all metrics, the reads-only strategy produced significantly better sensitivity than all other strategies, whilst the Flye assembly with or without Medaka polishing produced significantly better results than Miniasm for balanced accuracy and significantly better specificity than the reads-only strategy. The Medaka and reads-only strategies were, therefore, taken forward for more detailed analysis across all tools.

**Fig. 2.**
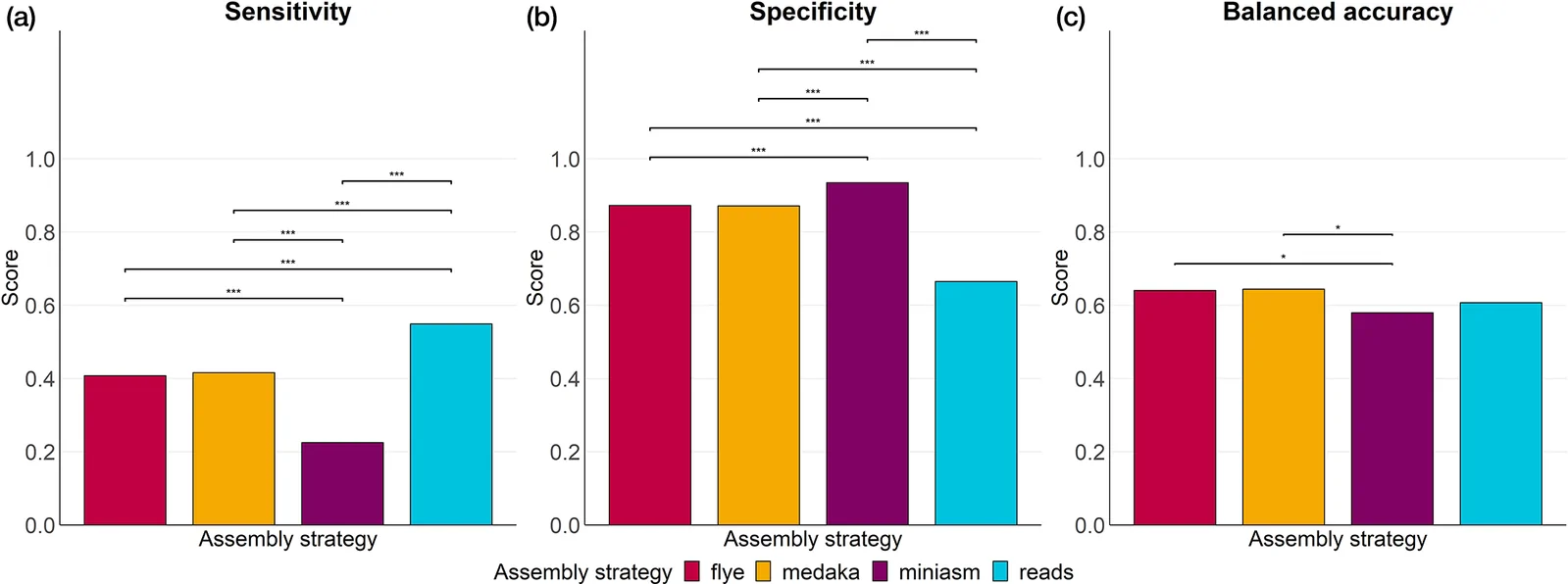
Overall performance metrics across 151 monoculture samples and all AMR detection tools, stratified by assembly strategy. (**a**) Sensitivity, (**b**) specificity and (**c**) balanced accuracy. ‘Flye’ refers to assembly with Flye in nano-hq mode, ‘medaka’ refers to assembly with Flye followed by polishing with Medaka, ‘miniasm’ refers to rapid assembly with Miniasm and ‘reads’ refers to prediction directly from raw sequencing reads without an assembly step. Of the tools tested here, only AMR++, DeepARG and ResFinder include a read-only mode. Statistical significance was calculated using the Kruskal–Wallis test and Dunn’s post-hoc test; *P*-values indicate pairwise significance. *** indicates *P*<0.0005, ** indicates *P*<0.005 and * indicates *P*<0.05.

### ResFinder-based tools produce the best overall balanced accuracy

The mean score for each performance metric across the 151 monocultured samples was calculated and stratified according to prediction strategy (tool, database, Medaka assembly vs reads) to determine the best performing tool overall. Fig. 3 shows the results for sensitivity, specificity and balanced accuracy.

**Fig. 3.**
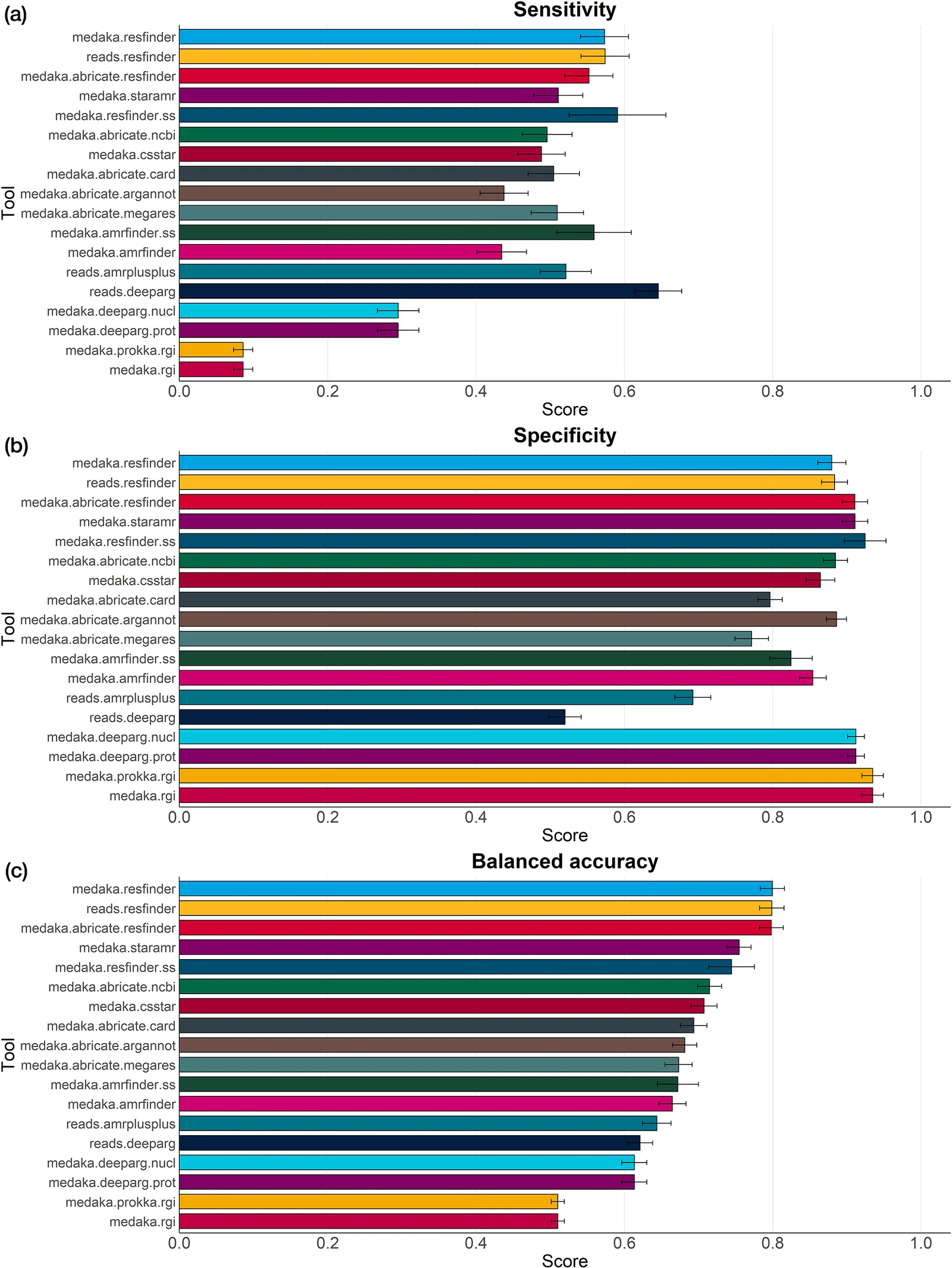
Performance metrics for each AMR prediction strategy, calculated at the level of specific antibiotics per sample then stratified by prediction strategy across the 151 monoculture samples. ‘Tool’ names follow the general structure assembly/reads.tool.(database).(ss), where (database) is only relevant for those tools with multiple database options and (SS) is only relevant for those tools with a species-specific mode. ‘reads’ indicates prediction directly from raw sequencing reads, whereas ‘medaka’ indicates Flye assembly followed by Medaka polishing. (**a**) shows the mean sensitivity, (**b**) shows the mean specificity and (**c**) shows the mean balanced accuracy. Prediction strategies are ordered by descending mean balanced accuracy, with the same order retained across all three panels. Error bars indicate se. Approximate 95% CIs and precision estimates for all prediction strategies are provided in Supplementary Table S13. Abbreviations: NCBI, National Center for Biotechnology Information; CARD, Comprehensive Antibiotic Resistance Database; ARG-ANNOT, Antibiotic Resistance Gene-ANNOTation; RGI, Resistance Gene Identifier; MEGARes, MEGARes antimicrobial resistance database; nucl, DeepARG’s nucleotide-only mode; prot, DeepARG’s annotation/protein mode.

For balanced accuracy, which considers both sensitivity and specificity, the top five strategies were all based on the ResFinder database. Read-based ResFinder achieved a mean balanced accuracy of 0.80 (∼95% CI: 0.77–0.83), with comparable results for assembly-based ResFinder and ABRicate using the ResFinder database and slightly lower results for StarAMR (Table S13).

Sensitivity scores were generally lower than specificity. The read-based DeepARG mode scored the highest at 0.65 (∼95% CI: 0.58–0.71). The lowest sensitivity scores were for both RGI strategies (assembly and annotations) at 0.09 (∼95% CI: 0.06–0.11), meaning that most AMR genes were not called.

Conversely, both RGI modes ranked the highest in specificity: 0.93 (∼95% CI: 0.91–0.96) for both assembly and annotation-based. However, as specificity relates to the absence of FP calls, RGI scoring highly does not mean that it performed particularly well; rather, RGI made the fewest calls simply because it made so few positive calls (false or true) in general. Read-based DeepARG performed best in sensitivity, but worst in specificity (0.52, ∼95% CI: 0.48–0.56), indicating a high number of apparent FPs alongside the TPs. In contrast, the two modes of the ResFinder tool score relatively well for specificity, at 0.88 (∼95% CI: 0.84–0.92).

### Using ‘species-specific’ databases may increase the accuracy of AMR predictions

We investigated the species-specific mode of two of the AMR tools tested here: ResFinder and AMRFinderPlus, to test whether using databases of species-specific genes and point mutations improved the overall accuracy of AMR predictions. Table S4 shows the species for which species-specific modes were available for the two tools. For ResFinder, species-specific predictions were only available for three of the species in our dataset (*n*=56 monoculture samples), whereas for AMRFinderPlus they were available for an additional 11 (14 total species, *n*=122 monoculture samples). Compared to the non-species-specific results, the sensitivity and specificity of species-specific ResFinder improved slightly (0.57 to 0.59 and 0.88 to 0.92, respectively). For AMRFinderPlus, the species-specific sensitivity showed a larger increase (0.43 to 0.56), but the specificity decreased (0.85 to 0.82).

### Making predictions based on antibiotic classes improves sensitivity, but reduces specificity

We tested whether grouping antibiotics into their classes (Table S4) produced better predictions, as some of the tools tested originally reported AMR at the class level only. Fig. 4 shows the per-class results for balanced accuracy, sensitivity and specificity, stratified by tool/database/reads vs assembly. The strategies which performed best at the level of specific antibiotic also performed best here: ResFinder-based tools were the top five scorers in balanced accuracy, whilst read-based DeepARG performed best in sensitivity but worst in specificity. Compared to predictions for specific antibiotics, read-based and assembly-based ResFinder scored worse for specificity: 0.88 per specific antibiotic and 0.81 per antibiotic class. In contrast, sensitivity was significantly improved: 0.57 per specific antibiotic and 0.73 per antibiotic class for ResFinder and 0.65 per antibiotic and 0.80 per class for DeepARG.

**Fig. 4.**
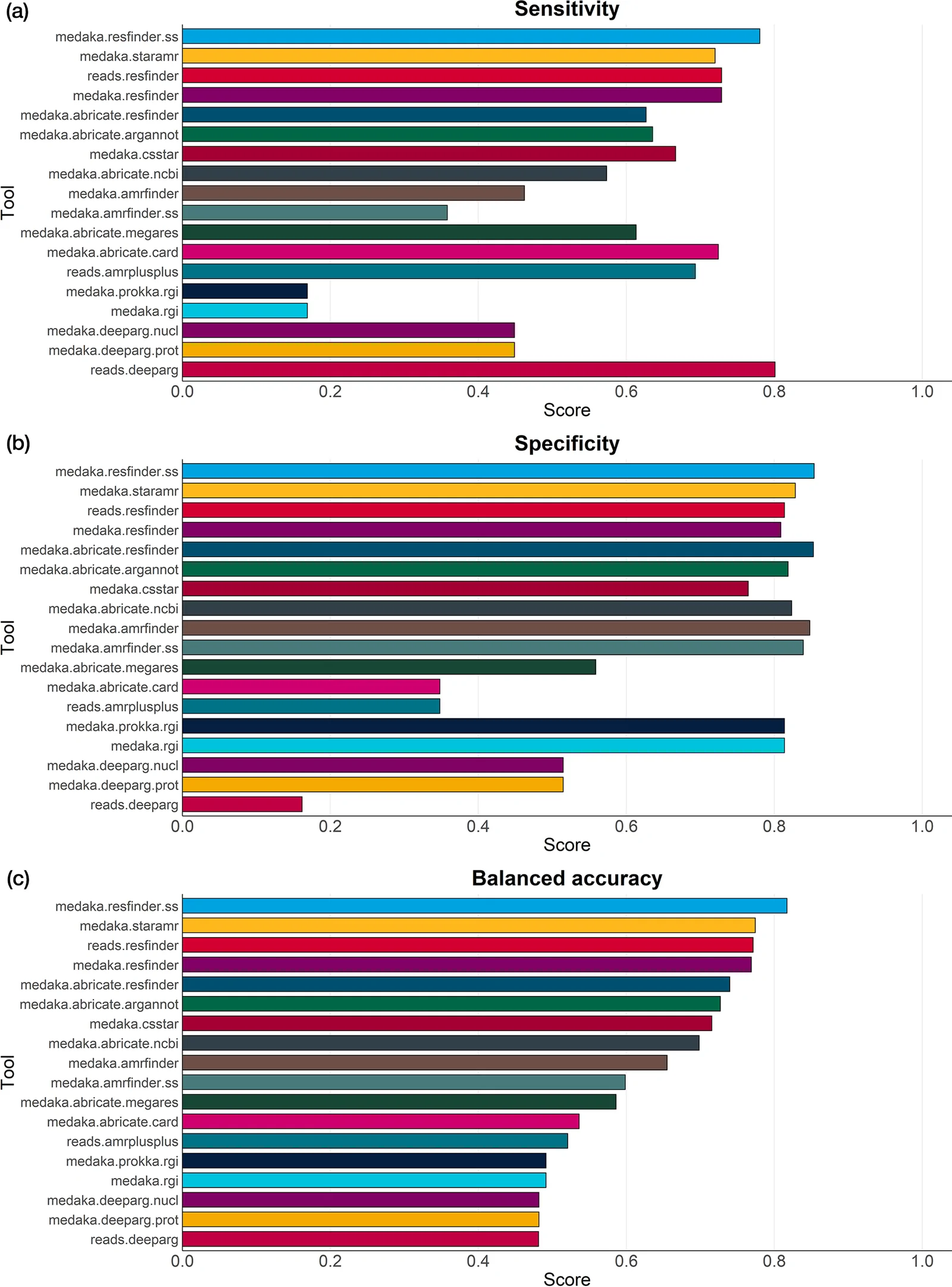
Performance metrics for each AMR prediction strategy, calculated at the level of antibiotic class and stratified by prediction strategy across the 151 monoculture samples. ‘Tool’ names follow the general structure assembly/reads.tool.(database).(ss), where (database) is only relevant for those tools with multiple database options and (SS) is only relevant for those tools with a species-specific mode. ‘reads’ indicates prediction directly from raw sequencing reads, whereas ‘medaka’ indicates Flye assembly followed by Medaka polishing. (**a**) shows the mean sensitivity, (**b**) shows the mean specificity and (**c**) shows the mean balanced accuracy. Prediction strategies are ordered by descending mean balanced accuracy, with the same order retained across all three panels. Error bars indicate se Abbreviations: NCBI, National Center for Biotechnology Information; CARD, Comprehensive Antibiotic Resistance Database; ARG-ANNOT, Antibiotic Resistance Gene-ANNOTation; RGI, Resistance Gene Identifier; MEGARes, MEGARes antimicrobial resistance database; nucl, DeepARG’s nucleotide-only mode; prot, DeepARG’s annotation/protein mode.

### AMR predictions are more accurate for some antibiotic classes than for others

We investigated whether AMR predictions for some classes were more robust than for others. Fig. 5 shows extensive significant differences in sensitivity, specificity and balanced accuracy across the different classes. Of note for sensitivity are polypeptides and fluoroquinolones, which performed particularly poorly (0.07 and 0.22, respectively) and aminoglycosides and tetracycline, which performed the best (0.69 and 0.72, respectively). Glycopeptides, although tested in only a small number of samples, performed well for specificity (0.93) but more poorly for sensitivity (0.52).

**Fig. 5.**
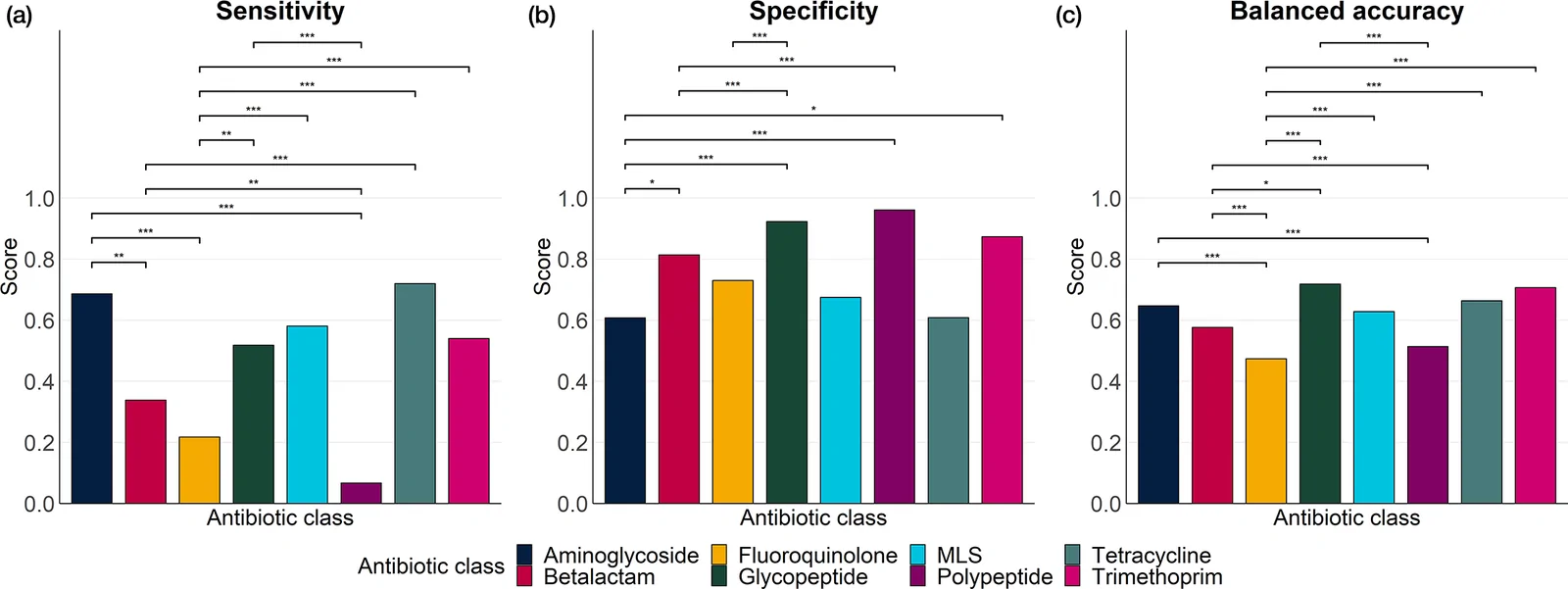
Overall performance metrics across 151 monoculture samples across all prediction strategies, stratified by antibiotic class. (**a**) Mean sensitivity, (**b**) mean specificity and (**c**) mean balanced accuracy. MLS indicates macrolide–lincosamide–streptogramin. Statistical significance was calculated using the Kruskal–Wallis test and Dunn’s post-hoc test; *P*-values indicate pairwise significance. *** indicates *P*<0.0005, ** indicates *P*<0.005 and * indicates *P*<0.05.

### AMR predictions are more accurate for some species than for others

To further investigate the factors affecting the accuracy of our predictions, we calculated the mean sensitivity, specificity and balanced accuracy across the 151 monocultured samples for the overall best performing prediction strategy (assembly with Flye, polishing with Medaka, prediction with ResFinder), stratified by the genus of the original sample. Fig. 6 shows that the predictions for some species are significantly more accurate than for others. *Klebsiella* (*n*=5) scored 100% for sensitivity, meaning every instance of AMR was predicted correctly, while *Staphylococcus* (*n*=36) also scored well, with almost 90% sensitivity. Meanwhile, both *Pseudomonas* (*n*=18), *Haemophilus* (*n*=8) and *Mycobacterium* (*n*=10) scored very poorly for sensitivity; every instance of AMR in *Mycobacterium* was missed. Most genera performed better for specificity than for sensitivity, with *Mannheimia* (*n*=15), *Enterococcus* (*n*=11), *Mycobacterium* (*n*=10) and *Neisseria* (*n*=7) all scoring 100% and *Escherichia* (*n*=44) and *Staphylococcus* (*n*=36) both scoring over 90%.

**Fig. 6.**
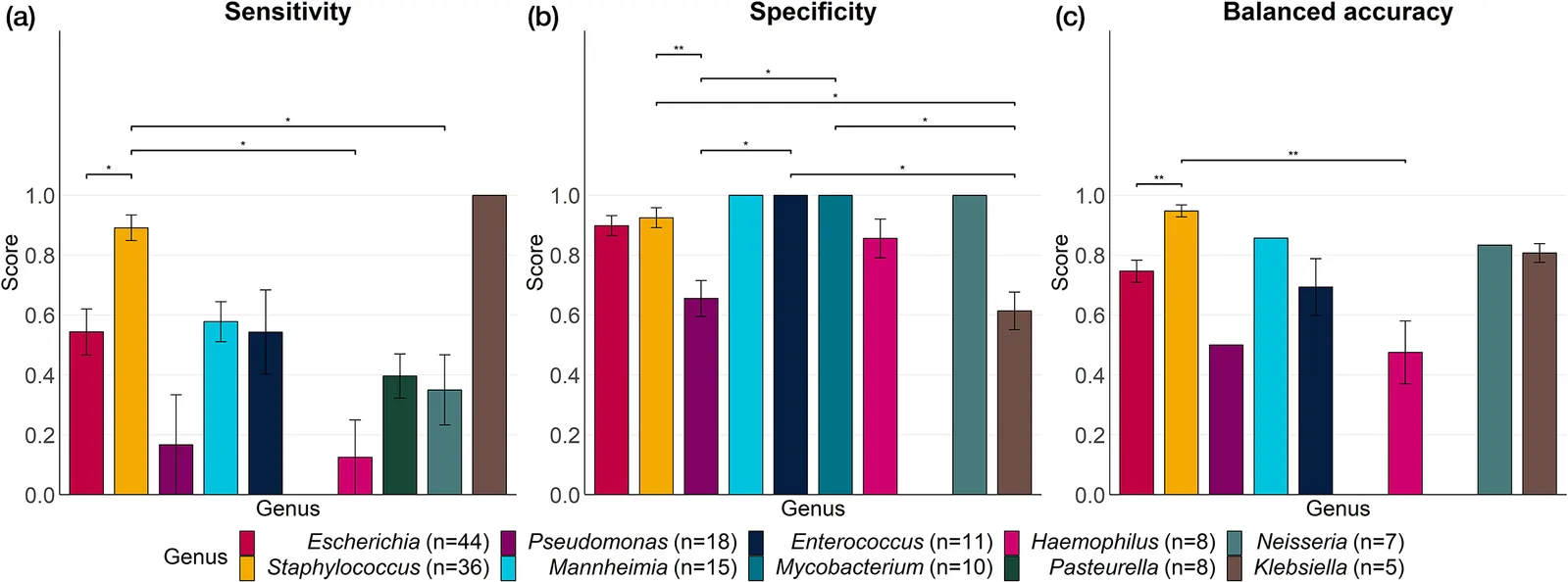
Overall performance metrics across 151 monoculture samples assembled with Flye, polished with Medaka and screened for AMR using ResFinder, stratified by genus identified in the original sample. (**a**) Mean sensitivity, (**b**) mean specificity and (**c**) mean balanced accuracy. Where a genus scored 0 in either sensitivity or specificity, balanced accuracy could not be calculated. Error bars show se. The legend indicates the number of samples included for each genus. Statistical significance was calculated using the Kruskal–Wallis test and Dunn’s post-hoc test; *P*-values indicate pairwise significance. *** indicates *P*<0.0005, ** indicates *P*<0.005 and * indicates *P*<0.05.

### AMR prediction tools for samples with mixed populations or host contamination perform similarly to more simple samples

To test how confounding factors, such as host DNA contamination, affected the prediction accuracy of our AMR tools, we processed 50 metagenomic/mixed species samples through the same analysis pipeline. This showed the same pattern as the monoculture results: ResFinder-based tools performed best for overall balanced accuracy whilst DeepARG performed best for sensitivity but worst for specificity. However, although the performance pattern remained the same, the actual scores observed for the ‘difficult’ samples were lower than those of the monocultured samples. Read-based ResFinder scored 0.75±0.02 for balanced accuracy compared to 0.80±0.02 for the monocultures, whilst DeepARG scored 0.58±0.03 for sensitivity compared to 0.65±0.03 for monocultures. Full results are available in the Supplementary Materials (Fig. S1).

### Consistency of performance patterns across sequencing conditions and dataset sources

As described, we assessed the consistency of the main performance patterns across different sequencing conditions and dataset compositions. Re-basecalling 64 monocultured isolate datasets using Dorado fast, HAC and SUP modes produced no statistically significant differences in sensitivity, specificity or balanced accuracy, although sensitivity showed a slight upward trend with increasing basecalling accuracy. Similarly, a separate species-matched comparison of 14 R9 and 14 R10 datasets detected no significant differences in these metrics. Stratification by original source study showed variation in performance estimates but broadly supported the tool/database-dependent sensitivity and specificity patterns observed in the pooled analysis (Table S14). Finally, the separate analysis of 50 mixed or host-contaminated datasets showed similar broad tool-performance patterns, albeit with lower absolute scores: read-based ResFinder achieved a balanced accuracy of 0.75±0.02 compared with 0.80±0.02 in the monocultured isolates, while DeepARG sensitivity was 0.58±0.03 compared with 0.65±0.03. Crucially, taking these findings together, we found no evidence that chemistry or basecaller materially drove the broad conclusions or performance patterns.

### Increasing starting data volume raises the overall accuracy of AMR predictions

The effect of data volume on the AMR prediction performance was assessed by analysing a subset of our samples filtered to 500, 250, 100, 50 and 25 Mb. Fig. 7 shows that a significant improvement is seen in the sensitivity and balanced accuracy of the AMR predictions from 25 to 500 Mb. There is an upward trend across all volumes in both sensitivity and balanced accuracy, although for both, the only statistically significant differences are between 25 Mb and each of the other volumes. The incremental improvements in sensitivity get smaller with additional data: the difference between 25 Mb and 50 Mb is 0.07 (0.38 to 0.45), compared to only 0.01 between 250 and 500 Mb (0.51 to 0.52). All significant differences seen across specificity were again between 25 Mb and the other volumes (except 50 Mb); in this case, there is a significant decrease from 25 to 500 Mb (0.88 to 0.82). This is likely due to overall fewer (true or negative) positive calls being made at lower data volumes due to poorer assembly quality. At higher data volumes, more positive calls are made, resulting in more TPs, but also more FPs.

**Fig. 7.**
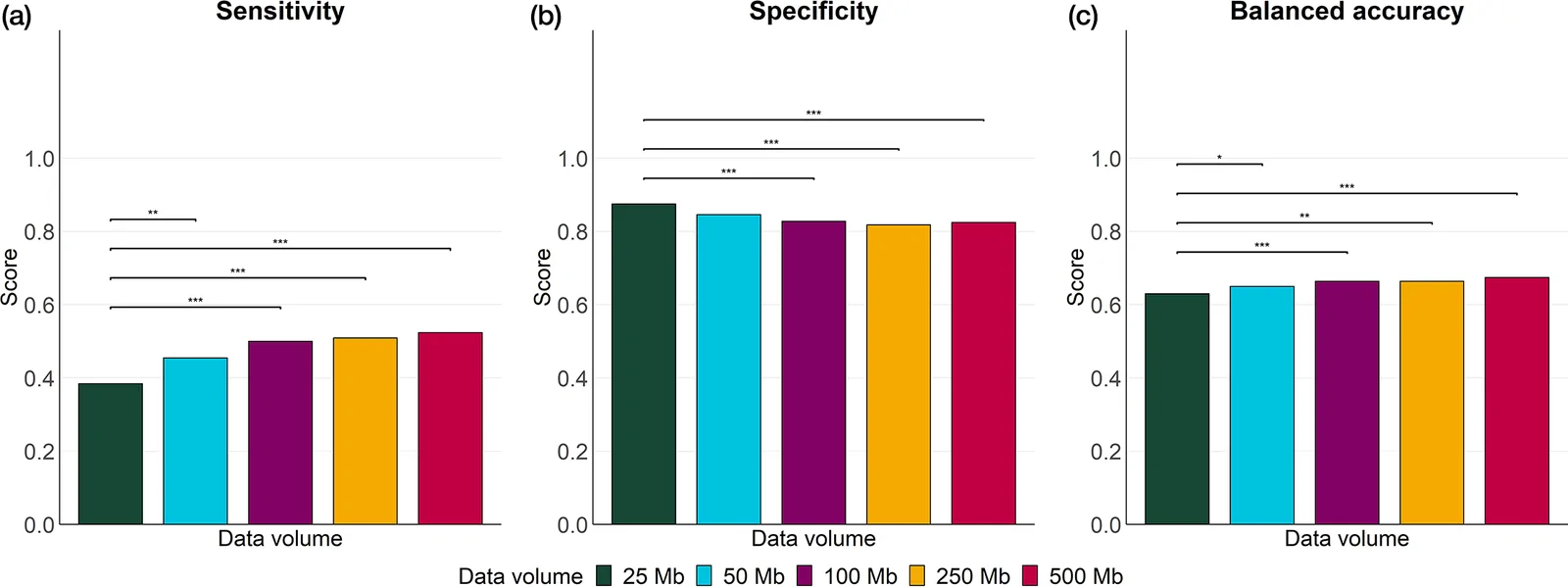
Performance metrics for the 61-sample subset filtered to different volumes of raw sequencing data using Filtlong, from 500 Mb down to 25 Mb. (**a**) Sensitivity, (**b**) specificity and (**c**) balanced accuracy. Metrics were calculated across all tools and samples, then stratified by starting data volume. Statistical significance was calculated using the Kruskal–Wallis test and Dunn’s post-hoc test; *P*-values indicate pairwise significance. For balanced accuracy, the Kruskal–Wallis test indicated a significant trend across all volumes, but no pairwise comparison was significant by Dunn’s post-hoc test. *** indicates *P*<0.0005, ** indicates *P*<0.005 and * indicates *P*<0.05.

## Discussion

### Benchmarking AMR prediction tool performance with ONT data could facilitate future clinical implementation and inform choice of tool for surveillance studies

In this study, we set out to determine how well existing computational tools can detect AMR in gDNA datasets derived from ONT sequencers, by comparing *in silico* AMR predictions (WGS-based antimicrobial susceptibility predictions, WGS-ASP) with paired *in vitro* AST results. Although there are many *in silico* AMR detection tools available, the majority were originally designed with Illumina short-reads in mind. Indeed, some commonly used tools (e.g. ARIBA [85], SRST2 [86], Groot [87]) could not be tested here, as they are for short-read data only. The new Amira tool is designed specifically for long-reads; however, at the time of testing it was only available for a limited number of species and thus we did not include it [88].

The tools which can be used with ONT data were still, therefore, originally optimized to detect AMR genes and mutations based on the characteristics of Illumina reads. These are short (<500 bp) reads with high (often >99.5%) raw accuracy, and any errors tending to occur as substitutions at the end of reads [89]. These tools, therefore, may not perform as well with reads from ONT sequencers, which are long (typically >1,000 bp) and historically had a higher raw error (1–5%), with errors tending to be insertions or deletions (indels) [90]. Raw accuracy is much higher using the latest ONT sequencing chemistry and basecaller (R10.4.1 flow cells and Dorado commonly produce raw error <1% [91]). However, our comparison of a small subset of species-matched samples sequenced on R10.4.1 and R9.4/R9.4.1 showed no significant difference between the overall accuracy of AMR predictions from the latest chemistry and basecaller and those of their predecessors. Tools designed with an Illumina error profile in mind may, therefore, have still resulted in missed AMR calls. For example, RGI reports Perfect and Strict hits by default, applying exact-match or CARD model-specific detection criteria; this may have contributed to its low sensitivity in our dataset as even minimal indels could cause frameshifts in the gene sequences, which would prevent their accurate translation into protein sequences, leading to FN results [35]. Applying RGI’s ‘--include_loose’ flag could allow for higher sensitivity with ONT data.

With metagenomic ONT sequencing likely to be used diagnostically in clinical settings, it will be vital that robust computational methods are used to identify species and detect AMR profiles. Accurately classifying AMR in gDNA datasets is also useful in other contexts, such as monitoring the environmental load of AMR-carrying bacteria within healthcare or One Health settings [92–95]. Whilst there are well-established and maintained guidelines for the interpretation of *in vitro* lab-based ASTs [96], no such guidelines currently exist for interpreting WGS-ASP. Understanding the strengths and weaknesses of the possible strategies to detect AMR from genomic data will, therefore, be important when determining which methods to implement in clinical settings. Whilst benchmarking has previously been done for short-reads [29] but there is less evidence for the accuracy of prediction strategies from ONT long-read sequencing data, although we note that a very recent publication from Govender *et al*. benchmarked several of the same tools tested here with metagenomic samples from blood cultures and also found that ResFinder produced the results most closely matched to AST [97]. In addition, we here address the impact of several important data steps prior to AMR prediction: namely, basecalling accuracy, volume of data and assembly strategy.

It should be acknowledged that the majority of our comparisons here were carried out using monocultured isolate samples (*n*=151); there were simply far more datasets for this kind of sample available. Although our separate analysis of the metagenomic samples (*n*=50) showed slight decreases in the performance metrics compared to the monoculture samples (for example, the decrease of ResFinder’s balanced accuracy from 0.80 to 0.75), we observed the same overall patterns in tool performance. The results of the monoculture analysis can, therefore, be regarded as a cautious proxy for more complex samples, as well as informing tool choice for isolate-based studies. We also note that in those mixed datasets exceeding 0.5 Gb, filtering to retain longer, higher-quality reads may also have reduced the representation of low-abundance organisms; this potential effect was not assessed separately.

In addition, both datasets also included uneven contributions from multiple source studies, which differed in sample number, sequencing chemistry, basecaller version and accuracy mode, genera and AST panels. The inclusion of older sequencing chemistries was important when considering the applicability of this benchmark: although R10.4.1 represents the most current technological advance over R9, R9-derived datasets remain relevant to both the reuse of existing publicly available sequencing data and contemporary clinical applications. A recent comparison of methods used by the NHS Respiratory Metagenomics Network reported sequencing on R9.4.1 flow cells using the Q-line GridION platform, which the authors described as being in current use throughout the UK-wide Network [98]. Restricting our benchmark to R10 data alone would, therefore, exclude datasets relevant to both existing research and current diagnostic workflows. Importantly, our species-matched comparison found no significant difference in AMR prediction performance between the R9- and R10-derived datasets, suggesting that inclusion of older sequencing data did not drive the broad conclusions of this study, although we note the relatively small scope of this comparison. Nonetheless, source study composition may have had some influence on our performance estimates and tool rankings, although the source-stratified findings (Table S14) supported the main interpretation of the pooled analysis: performance varied by tool/database and reflected sensitivity/specificity trade-offs.

### Choice of basecalling mode does not significantly affect AMR prediction accuracy, but choice of assembly strategy does

Before comparing the performance of the AMR prediction tools and databases, we optimized the strategy for preparing sample data. The first step in analysing ONT data is basecalling, i.e. translating the raw electrical signal (the ‘squiggle’) into a DNA sequence. Early basecallers, such as Albacore, which was used to basecall 34 of the samples in our dataset, did not have different basecalling modes. However, Guppy and Dorado have three accuracy modes: fast, HAC and SUP. SUP basecalling requires much higher computing resources and takes an order of magnitude longer than fast basecalling, albeit with the benefit of significantly increased per-base accuracy in the resulting FASTQ reads. HAC basecalling is the middle-ground option between fast and SUP. ONT does not currently publish an expected read accuracy for fast basecalling, but quotes a median raw read accuracy of 99.4 and 99.7%, respectively for the latest HAC and SUP models [99].

SUP basecalling within a rapid diagnostic timeframe would require a GPU-powered computer or GridION, which may not be economically viable in all clinical settings. However, despite a slight trend in our results towards more accurate WGS-ASP from fast to HAC to SUP, the differences were not significant. Our results, therefore, indicate that even fast basecalling, which is feasible using a standard laptop with 2–4 CPUs and 8–16 GB RAM, may produce AMR predictions with comparable accuracy to HAC or SUP basecalling. One possible explanation for the limited improvements seen from fast to SUP is that improvements in per-base sequence accuracy do not necessarily change whether an AMR determinant exceeds a tool’s detection thresholds. For example, ABRicate and ResFinder used minimum sequence identity thresholds of 80%; improving the accuracy of a sequence already exceeding this threshold may not alter the resulting presence/absence call. This same explanation could also apply to the limited differences seen between the R9 and R10 results. In assembly-based workflows, consensus generation may also reduce the effect of read-level errors. However, this explanation is unlikely to apply uniformly, as some tools use more stringent or model-specific detection criteria, and the detection of resistance-conferring point mutations will remain sensitive to individual nucleotide errors. We note also that our basecalling analysis was based on monocultured isolates; it is possible, therefore, that different basecalling accuracy modes may produce more significantly different results when applied to metagenomic samples.

The next step in data preparation after basecalling is assembly into contigs. A previous comparison of AMR prediction tools focusing on short-read data included tools that analysed reads but not assembled contigs [29]. Although assembling reads into contigs requires additional processing steps and, therefore, additional time, the processing of individual reads into a consensus sequence greatly improves the per-base accuracy [100]. For ONT data, which may be less accurate than Illumina data at the read level, computing a consensus may, therefore, result in more accurate AMR predictions. Our results show that Miniasm, which exchanges accuracy for speed, performed the worst overall in sensitivity and balanced accuracy. Flye and Flye+Medaka produced results that were not significantly different from each other; this finding is unsurprising, since newer sequencing chemistries and basecallers may not need post-assembly polishing [101]. Because full basecalling model metadata was not available for all samples in our dataset, we used Medaka’s automatic model-selection mode; in some cases, Medaka may have been unable to identify the basecalling model from the input data itself, meaning the default consensus model would have been used. As the specific Medaka model selected for each dataset was not recorded, we cannot exclude potential effects of the automated model selection on individual assemblies. Nevertheless, the absence of significant differences in overall AMR prediction performance between Flye assemblies with and without Medaka polishing suggests that polishing was not a major determinant of the broad performance patterns observed here. As Fig. 2 does show a marginal improvement from Flye to Flye+Medaka, we chose to use the Medaka assemblies instead of Flye when analysing the performance of each AMR prediction tool separately.

### ResFinder database produces results which most closely match AST, whilst DeepARG produces the highest sensitivity

We tested a variety of tools with a variety of databases, in both assembly and read-based modes. Where a tool allowed either assembly or read mode to be used, we trialled both. Likewise, where a tool allowed the selection of AMR databases (e.g. ABRicate), we trialled all available. ResFinder, CARD, MEGARes and NCBI’s AMRFinderPlus/NDARO (National Database of Antibiotic Resistant Organisms) are among the most commonly used AMR gene databases [102], and all were trialled with more than one tool. Our results show that, in terms of balanced accuracy between sensitivity and specificity, the ResFinder database produces WGS-ASP which most closely match the AST results, independent of the tool used to screen the database. Of the ResFinder-based tools, the standalone ResFinder tool performed best.

ResFinder performed almost exactly the same when used with assembled contigs or with raw reads, suggesting that important AMR information from the reads is not lost during consensus building. ResFinder performed best in species-specific mode, although the ResFinder command line tool only includes species-specific screening for a very limited number of species; here, we could only test it with *E. coli*, *Enterococcus faecalis* and *Enterococcus faecium* (*n*=56). Notably, the ResFinder webserver [103] offers species-specific screening with PointFinder for far more species than the command-line tool. In addition, the webserver is user-friendly and outputs an easily interpreted results report. One AMR prediction strategy for rapid diagnostics would, therefore, be to simply use the ResFinder webserver with either assembled FASTA or raw FASTQ reads, selecting any species known to be in the sample. However, this relies on a shared public web service; analysis turnaround time may, therefore, vary with server load and concurrent usage. In addition, the need to upload genetic data to an online tool could raise ethical concerns, particularly when working with metagenomic samples originally from humans, which may still contain human DNA sequences.

Meanwhile, DeepARG with raw reads displayed the highest sensitivity here, albeit with the lowest specificity. DeepARG’s relatively permissive minimum alignment identity threshold (50%), together with its deep-learning classification approach which applies a separate prediction-probability threshold of 0.8, may facilitate the detection of more divergent AMR determinants and contribute to its higher sensitivity. However, broader detection may also increase the number of apparent false-positive calls relative to phenotypic AST, contributing to its lower specificity. Although the results of DeepARG are not the closest match to the AST results, therefore, DeepARG detects the overall highest number of AMR genes and may represent a strong choice for surveillance of the full acquired resistance gene load in metagenomic samples.

### How accurate are the results of ‘gold standard’ tests?

AST remains the most appropriate reference standard for benchmarking *in silico* AMR prediction, because it directly measures the resistance phenotype *in vitro*. However, phenotypic susceptibility testing is not error-free, with reproducibility and other outcomes ranging from 90–99% [104]. Phenotypic AST results can vary with method, inoculum, media, incubation conditions, endpoint reading and breakpoint interpretation; thus, single AST results should not be interpreted as a fixed ‘ground truth’, a point which was emphasized in the recent European Committee on Antimicrobial Susceptibility Testing (EUCAST) update on WGS-ASP [105, 106]. Variability close to clinical breakpoints is particularly important, and small technical or biological differences can alter whether an isolate is categorized as susceptible, intermediate or resistant.

Consequently, although FP or FN results may reflect real errors by the prediction tool, some apparent FP or FN *in silico* predictions may instead occur due to limitations in the AST methods used, with limited ability to distinguish between the two. Isolates with known resistance determinants can, therefore, be deemed susceptible by ASTs, because a gene is present, but not phenotypically expressed at levels deemed ‘resistant’ under laboratory conditions [107, 108]. Such ‘silent’ or poorly expressed resistance genes still reflect a latent AMR potential that could be clinically relevant, particularly if expression is induced *in vivo* or under antibiotic selection pressure.

Genotype–phenotype discordance may also arise when a detected resistance determinant is incomplete or functionally altered. For example, Ivanova *et al*. demonstrated that azithromycin susceptibility among mph(A)-positive isolates was associated with disruptions to the wider operon and regulatory region, while Iurescia *et al*. identified cfr-positive, linezolid-susceptible isolates carrying a frameshift-causing deletion [109, 110]. These findings highlight the importance of gene-specific sequence identity, completeness and genomic context for accurate phenotype prediction. Consequently, differences in the detection thresholds and reference databases used by AMR prediction tools may contribute to apparent false-positive and false-negative calls. In this study, we compared tools using their documented settings rather than optimizing detection thresholds for individual resistance determinants.

These sources of discordance may affect the apparent performance of all tools evaluated here, particularly because our datasets were gathered from many different original studies which may have applied different AST methods in different laboratory settings. In particular, tools or databases that make broader genotype-to-phenotype assignments may generate more apparent FPs relative to AST. If these ‘FP’ calls are actually due to variability in the way AST breakpoints were interpreted, or under-expression of genes in laboratory conditions, a tool with apparently poor specificity may, therefore, actually represent a more conservative option for complementing clinical decisions.

Handling of ‘intermediate’ resistance calls can also introduce apparent genotype-phenotype discordance. The EUCAST guidelines no longer group intermediate calls with resistant calls; instead, intermediate calls are treated as ‘susceptible, but requires longer exposure’, although it has been argued that this increases the rates of both FP and FN AST calls [111, 112]. Here, we grouped resistant and intermediate phenotypes for the purposes of benchmarking genotype-based detection of reduced susceptibility, treating only ‘susceptible’ phenotypes as truly susceptible. This means that if a tool detected no AMR determinants in an ‘intermediate’ sample, we would deem this a FN, but under the EUCAST guidelines it would be called susceptible, and, therefore, a TN. Nonetheless, as only 20 of the 1,427 AST calls analysed here were ‘intermediate’, the classification of these as either resistant or susceptible likely had minimal impact on our overall results.

Therefore, although AST was retained as the ‘ground truth’ for this benchmarking study, performance metrics should be interpreted as agreement with the available phenotypic test results, rather than as an absolute measure of biological truth.

### Is *in silico* AMR prediction ready for use in a clinical setting with ONT data?

In this study, we identified an optimal strategy for preparing ONT data for AMR prediction and compared the performance of AMR prediction tools and databases. Tools using the ResFinder database strike the best balance between sensitivity and specificity, while DeepARG performs the best in terms of sensitivity. However, even the best-performing tool missed some AMR reported by phenotypic AST.

Although no diagnostic test is perfect, in order for a test to be trusted for making clinical decisions, its accuracy would ideally approach 100% [113]. DeepARG, the best tool for sensitivity, detected only 65% of the AMR in our AST results. A number of factors may have led to this. Our dataset represented a wide variety of sequencing qualities, with some samples dating back to 2019 or earlier, sequenced on less accurate flow cells and basecalled with less accurate algorithms. However, our results suggest that neither basecalling accuracy nor sequencing chemistry significantly affected the accuracy of WGS-ASP. Conversely, some of our samples had minimal data, and our data volume test shows that even 500 Mb might not capture all AMR present in a sample.

Most importantly, a variety of genera were included in our samples. We included these to ensure our testing was representative of the spectrum of pathogens that could be present in clinical mWGS samples. Our results showed that whilst some genera scored highly for sensitivity, specificity and balanced accuracy, others scored poorly, particularly for sensitivity. The genera which scored the worst for sensitivity included those known to perform poorly with standard acquired resistance gene databases, such as *Pseudomonas*, in which resistance is usually a result of chromosomal variants, efflux, porin changes and regulatory mechanisms and *Haemophilus*, which accumulates resistance almost entirely through single nucleotide polymorphisms and indels [114, 115]. *Mycobacterium* in particular is known to acquire resistance using mechanisms not usually represented in standard gene databases, and prediction of AMR tends to require the use of *Mycobacterium*-specific tools, such as TBProfiler [116–118]. We may conclude, therefore, that the overall performance of the tools analysed here, which largely did not include mutation-based detection, has been influenced by the inclusion of genera for which gene-based AMR detection is difficult; for those genera whose resistance is based mainly on acquired genes, such as *Klebsiella*, the predictions from the tools tested here are much more reliable.

Predicting AMR based on antibiotic class instead of a specific antibiotic may lead to better sensitivity; our results showed an improvement from 65–80% for DeepARG. Using this strategy, if a gene was detected which was related to AMR for a specific antibiotic, all other antibiotics from that same class would automatically be excluded. For example, if a gene related to gentamicin resistance was detected, all aminoglycosides would be excluded and an entirely different antibiotic class would need to be used. However, predictive antibiotic susceptibility is variable between classes, bacterial species and AMR genes. In our study, performance for aminoglycosides, glycopeptides, tetracyclines and trimethoprim was reasonably good, whilst that for β-lactams was less impressive, and performance for polypeptides and fluoroquinolones was very poor. In genomic terms, this may reflect both gaps in AMR databases as well as resistance based on mutations rather than acquired genes (as seen in fluoroquinolone resistance) [119]. Most tools do not offer a species-specific mode or the option to screen for point mutations and those that do (such as ResFinder) offer it only for a limited group of species. Consequently, sensitivity to AMR caused by point mutations will be low. Care should, therefore, be taken with any class-based strategy to avoid excluding antibiotics that may be effective, or including those where resistance is likely. This is particularly important in serious infections and/or multi-drug-resistant scenarios where clinical outcomes depend on a rapid and accurate choice of antibiotic, and fewer antibiotics are available.

## Conclusions

We have here detailed several important findings for choosing an optimal pipeline for AMR prediction from nanopore data: choice of basecaller accuracy mode seems to exert little influence on overall WGS-ASP accuracy; increasing data volume results in more accurate WGS-ASP but with diminishing returns, and a strategy using either raw reads or contigs assembled with Flye (with or without Medaka polishing) will produce WGS-ASP with similar accuracy.

Taken together, our findings provide several practical considerations for researchers and diagnostic laboratories selecting workflows for AMR prediction from ONT sequencing data (Table 3).

**Table 3.** Practical implications of this study’s results

Sequencing variable	Findings from this study	Practical considerations
Sequencing chemistry (R9, R10, etc.)	No significant differences in AMR prediction performance were detected between R9 and R10 data in the species-matched subset.	Existing R9 datasets remain suitable for inclusion in downstream AMR analyses; the use of older sequencing chemistry alone does not necessarily preclude accurate prediction.
Basecalling accuracy (fast, HAC, SUP)	Fast, HAC and SUP basecalling produced no significant differences in AMR prediction performance across the tested monocultured isolates.	More computationally intensive basecalling may not provide a corresponding improvement in downstream AMR prediction. Fast or HAC basecalling may, therefore, be appropriate where computational resources or turnaround time are limiting.
Assembly strategy (reads, fast assembly, slower assembly, polishing)	Flye assemblies, with or without Medaka polishing, produced higher balanced accuracy than Miniasm. Read-based approaches produced higher sensitivity across the tools supporting this input type.	Flye-based assembly provides a suitable approach for assembly-dependent tools. Read-based prediction may be advantageous where sensitivity is prioritized and the selected tool supports direct read input.
AMR prediction tool/database	ResFinder-based approaches produced the highest observed balanced accuracy, while read-based DeepARG produced the highest sensitivity but lowest specificity.	Tool selection should reflect the intended application. ResFinder-based approaches provide a balance between detecting resistance and avoiding apparent false-positive predictions, whereas DeepARG may be useful for broader resistome screening.
Species-specific prediction	Performance varied substantially between genera, and species-specific prediction improved some performance metrics. Gene-centric approaches performed poorly for organisms with primarily mutation-mediated resistance mechanisms.	Species-specific and mutation-aware prediction should be considered where available. Absence of detected resistance genes should not necessarily be interpreted as evidence of susceptibility, particularly for organisms, such as *Pseudomonas* and *Mycobacterium*.
Sequencing data volume	Increasing data volume improved sensitivity and balanced accuracy, with smaller incremental improvements at higher volumes. Specificity decreased as additional positive predictions were made.	Sufficient sequencing data are needed to avoid missed resistance determinants, but increasing data volume alone does not guarantee improved overall prediction performance. A universal minimum sequencing depth cannot be established from these results.
Sample complexity and clinical interpretation	Broad tool performance patterns were similar in the separately analysed mixed/host-contaminated datasets, although absolute performance was lower than in monocultured isolates.	Findings from isolate sequencing can be extrapolated cautiously to complex clinical samples. Genomic AMR prediction can support phenotypic AST but will not necessarily provide complete concordance with it.

However, our results indicate that limitations remain for users considering the implementation of AMR prediction tools and databases with nanopore long-reads in clinical settings. This finding is consistent with an earlier study with Illumina short-reads and the recommendations of the recent EUCAST WGS-ASP report [29, 106]. Universal guidelines for how *in silico* predictions should be interpreted for each gene, species, antibiotic and antibiotic class are needed. Species-specific tools and databases may be required and indeed already exist for species like *Mycobacterium tuberculosis* and, increasingly, *Pseudomonas aeruginosa* [114, 116, 117, 120].

In the meantime, the results of *in silico* predictions should be used with caution, with clinical decisions being made on a case-by-case basis, taking into account the case history, pathogen species, antibiotic class and available drugs and bearing in mind that prediction tools work better for some bacteria and some antibiotics than for others. For example, a prediction of vancomycin (glycopeptide) resistance in *Staphylococcus* could be regarded with strong confidence, whereas the absence of detection of fluoroquinolone resistance in *Pseudomonas* would not necessarily reliably suggest susceptibility. Overall, based on our findings, we suggest that *in silico* AMR prediction using mWGS is ready to support and augment the results of *in vitro* AST, particularly in rapid patient-side diagnosis and treatment decisions, but it is not yet ready to replace it.

## Supplementary material

10.1099/mgen.0.001885 Supplementary Material 1.

10.1099/mgen.0.001885 Supplementary Material 2.

## Abbreviations

AMR: antimicrobial resistance
ARG-ANNOT: Antibiotic Resistance Gene-ANNOTation
AST: antimicrobial sensitivity testing
CARD: Comprehensive Antibiotic Resistance Database
CI: confidence interval
CPU: cental processing unit
DB: database
EUCAST: European Committee on Antimicrobial Susceptibility Testing
FN: false negative
FP: false positive
gDNA: genomic DNA
GPU: graphics processing unit
HAC: high accuracy
indels: insertions or deletions
MIC: minimum inhibitory concentration
MLS: macrolides–lincosamides–streptogramins
mWGS: metagenomic whole-genome sequencing
NCBI: National Center for Biotechnology Information
NDARO: National Database of Antibiotic Resistant Organisms
ONT: Oxford Nanopore Technologies
QC: quality control
RGI: resistance gene identifier
SD: standard deviation
SE: standard error
SRA: sequence read archive
ss: species-specific
SUP: super accuracy
TN: true negative
TP: true positive
WGS: whole-genome sequencing
WGS-ASP: whole-genome sequencing-based antimicrobial susceptibility prediction
